# 2-(Phenyl­sulfan­yl)pyridine-3-carboxylic acid

**DOI:** 10.1107/S1600536809038586

**Published:** 2009-10-07

**Authors:** Muhammad Naeem Khan, Misbahul Ain Khan, Muhammad Nadeem Arshad, Islam Ullah Khan, Salma Rehman

**Affiliations:** a Department of Chemistry, Islamia University, Bahawalpur, Pakistan; b Materials Chemistry laboratory, Department of Chemistry, GC University, Lahore, Pakistan; c Applied Chemistry Research Center, PCSIR Laboratories Complex, Lahore 54600, Pakistan

## Abstract

The title compound, C_12_H_9_NO_2_S, belongs to the nitro­gen-containing group of heterocyclic organic compounds and crystallized with two mol­ecules per asymmetric unit. In the crystal, both molecules form inversion dimers linked by pairs of O—H—O hydrogen bonds. Weak symmetry-related C—H—O inter­actions link the carboxyl dimers along *b* axis. The dihedral angle between the two aromatic rings in the two mol­ecules are 55.75 (14) and 58.33 (13)°.

## Related literature

For the pharmacological effects of heteroaromatic anti­tumor compounds: Denny *et al.* (1982 ▶); Fujiwara (1997 ▶); Antonini & Martelli (1992 ▶); Cholody *et al.* (1992 ▶). For the title compound as an inter­mediate for heterocycles, see: Khan *et al.* (2008*a* ▶,*b* ▶). For the synthesis, see: Mann & Reid (1952 ▶).
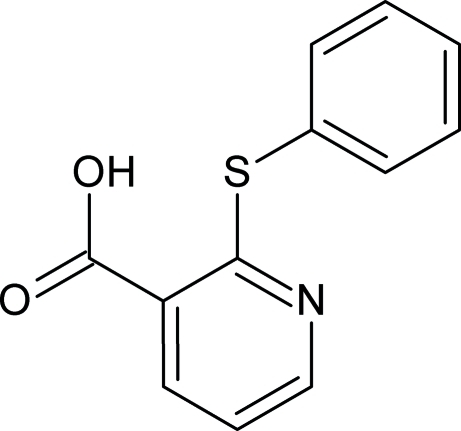


## Experimental

### 

#### Crystal data

C_12_H_9_NO_2_S
*M* *_r_* = 231.26
Triclinic, 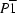

*a* = 7.2201 (4) Å
*b* = 7.6653 (4) Å
*c* = 19.9537 (11) Å
α = 97.895 (3)°
β = 98.520 (3)°
γ = 91.661 (3)°
*V* = 1080.41 (10) Å^3^
*Z* = 4
Mo *K*α radiation
μ = 0.28 mm^−1^
*T* = 296 K
0.21 × 0.09 × 0.06 mm

#### Data collection

Bruker Kappa APEXII CCD diffractometer
Absorption correction: multi-scan (*SADABS*; Bruker, 2005 ▶) *T*_min_ = 0.911, *T*_max_ = 0.983
23200 measured reflections
5397 independent reflections
2766 reflections with *I* > 2/s(*I*)
*R*_int_ = 0.063

#### Refinement

*R*[*F* ^2^ > 2σ(*F* ^2^)] = 0.073
*wR*(*F* ^2^) = 0.224
*S* = 1.09
5397 reflections
292 parameters
H-atom parameters constrained
Δρ_max_ = 0.59 e Å^−3^
Δρ_min_ = −0.53 e Å^−3^

### 

Data collection: *APEX2* (Bruker, 2007 ▶); cell refinement: *SAINT* (Bruker, 2007 ▶); data reduction: *SAINT*; program(s) used to solve structure: *SHELXS97* (Sheldrick, 2008 ▶); program(s) used to refine structure: *SHELXL97* (Sheldrick, 2008 ▶); molecular graphics: *ORTEP-3 for Windows* (Farrugia, 1997 ▶) and *PLATON* (Spek, 2009 ▶); software used to prepare material for publication: *WinGX* (Farrugia, 1999 ▶) and *PLATON*.

## Supplementary Material

Crystal structure: contains datablocks I, global. DOI: 10.1107/S1600536809038586/hg2570sup1.cif

Structure factors: contains datablocks I. DOI: 10.1107/S1600536809038586/hg2570Isup2.hkl

Additional supplementary materials:  crystallographic information; 3D view; checkCIF report

## Figures and Tables

**Table 1:** Hydrogen-bond geometry (Å, °)

*D*—H⋯*A*	*D*—H	H⋯*A*	*D*⋯*A*	*D*—H⋯*A*
O2—H2⋯O3^i^	0.82	1.82	2.624 (2)	167 (1)
O4—H4o⋯O1^i^	0.82	1.83	2.642 (2)	170 (1)
C3—H3⋯O4^ii^	0.93	2.50	3.264 (5)	139
C4—H4⋯O1^iii^	0.93	2.55	3.458 (5)	164
C15—H15⋯O2^ii^	0.93	2.54	3.294 (5)	138
C16—H16⋯O3^iii^	0.93	2.58	3.467 (5)	160

Symmetry codes: (i) 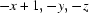; (ii) 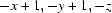; (iii) 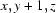.

**Fig. 1.**
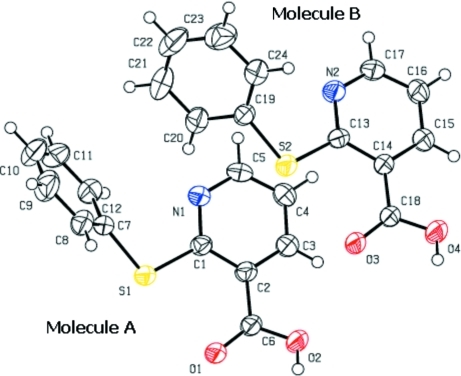
The molecular structure diagram of the title compound showing the atom labels. Thermal ellipsoids are drawn at the 50% probability level.

**Fig. 2.**
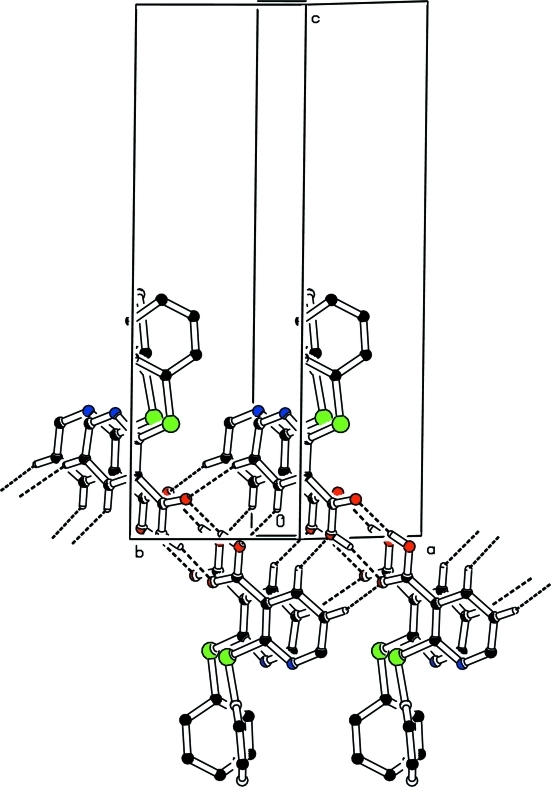
Unit cell packing diagram showing the intermolecular hydrogen bonding using dashed lines. The hydrogen atoms not involved in hydrogen bonding have been omitted.

## supplementary crystallographic information

### Comment

In continuation of our studies on pyridine-containing heterocyclic compounds, the title compond was synthesized. It is an intermediate for our previously reported crystal structures of 7-nitro-5*H*-thiochromeno[2,3-*b*]pyridin-5-one (Khan *et al.*, 2008*a*) and 5*H*-thiochromeno[2,3-*b*]pyridin-5-one (Khan *et al.*, 2008*b*). Pyridine containing compounds are widely distributed in nature. Heteroaromatic antitumor compounds have been prepared in recent years with the hope of increasing pharmacological effects (Denny *et al.*, 1982), (Fujiwara, 1997), (Antonini & Martelli, 1992) (Cholody *et al.*, 1992).

The title compound was crystallized with two independent molecules in the asymmetric unit (Fig 1). The dihedral angles between the two aromatic rings in molecule A and molecule B are 55.75 (14)° and 58.33 (13)° respectively. The carboxylic group present in each molecule forms dimers which are linked through weak C–H—O type interaction along the *b* axis to stabilize the structure Table. 1 & Fig. 2.

### Experimental

A mixture of 2-chloronicotinic acid (1.57 g, 10 mmol) and thiophenol (2 ml) was heated under reflux for two hours to produce 2-(Phenylsulfanyl)pyridine-3-carboxylic acid (Mann & Reid, 1952). Suitable crystals for X-ray diffractions were obtained on cooling the saturated solution of (I) in ethanol.

### Refinement

The H-atoms for aromatic carbons and carboxylic O atoms were refined geometrically and treated as riding atoms: C—H = 0.93Å with *U*_iso_(H) = 1.2 and O—H = 0.82 with *U*_iso_(H) = 1.5.

### Crystal data

**Table:**

C_12_H_9_NO_2_S	*Z* = 4
*M**_r_* = 231.26	*F*(000) = 480
Triclinic, *P*1	*D*_x_ = 1.422 Mg m^−^^3^
Hall symbol: -P 1	Mo *K*α radiation, λ = 0.71073 Å
*a* = 7.2201 (4) Å	Cell parameters from 3133 reflections
*b* = 7.6653 (4) Å	θ = 2.9–23.8°
*c* = 19.9537 (11) Å	µ = 0.28 mm^−^^1^
α = 97.895 (3)°	*T* = 296 K
β = 98.520 (3)°	Needle, white
γ = 91.661 (3)°	0.21 × 0.09 × 0.06 mm
*V* = 1080.41 (10) Å^3^	

### Data collection

**Table:**

Bruker Kappa APEXII CCD diffractometer	5397 independent reflections
Radiation source: fine-focus sealed tube	2766 reflections with *I* > 2/s(*I*)
graphite	*R*_int_ = 0.063
φ and ω scans	θ_max_ = 28.4°, θ_min_ = 1.0°
Absorption correction: multi-scan (*SADABS*; Bruker, 2005)	*h* = −9→9
*T*_min_ = 0.911, *T*_max_ = 0.983	*k* = −10→10
23200 measured reflections	*l* = −26→26

### Refinement

**Table:**

Refinement on *F*^2^	Secondary atom site location: difference Fourier map
Least-squares matrix: full	Hydrogen site location: inferred from neighbouring sites
*R*[*F*^2^ > 2σ(*F*^2^)] = 0.073	H-atom parameters constrained
*wR*(*F*^2^) = 0.224	*w* = 1/[σ^2^(*F*_o_^2^) + (0.1023*P*)^2^ + 0.1581*P*] where *P* = (*F*_o_^2^ + 2*F*_c_^2^)/3
*S* = 1.09	(Δ/σ)_max_ < 0.001
5397 reflections	Δρ_max_ = 0.59 e Å^−^^3^
292 parameters	Δρ_min_ = −0.52 e Å^−^^3^
0 restraints	Extinction correction: *SHELXL97* (Sheldrick, 2008), Fc^*^=kFc[1+0.001xFc^2^λ^3^/sin(2θ)]^-1/4^
Primary atom site location: structure-invariant direct methods	Extinction coefficient: 0.045 (5)

### Special details

**Table:**

Geometry. All e.s.d.'s (except the e.s.d. in the dihedral angle between two l.s. planes) are estimated using the full covariance matrix. The cell e.s.d.'s are taken into account individually in the estimation of e.s.d.'s in distances, angles and torsion angles; correlations between e.s.d.'s in cell parameters are only used when they are defined by crystal symmetry. An approximate (isotropic) treatment of cell e.s.d.'s is used for estimating e.s.d.'s involving l.s. planes.
Refinement. Refinement of *F*^2^ against ALL reflections. The weighted *R*-factor *wR* and goodness of fit *S* are based on *F*^2^, conventional *R*-factors *R* are based on *F*, with *F* set to zero for negative *F*^2^. The threshold expression of *F*^2^ > σ(*F*^2^) is used only for calculating *R*-factors(gt) *etc*. and is not relevant to the choice of reflections for refinement. *R*-factors based on *F*^2^ are statistically about twice as large as those based on *F*, and *R*- factors based on ALL data will be even larger.

### Fractional atomic coordinates and isotropic or equivalent isotropic displacement parameters (Å^2^)

**Table:**

	*x*	*y*	*z*	*U*_iso_*/*U*_eq_	
S1	0.15013 (14)	−0.12613 (12)	0.21464 (5)	0.0407 (3)	
S2	0.70162 (15)	0.36894 (12)	0.22267 (5)	0.0447 (3)	
O1	0.1649 (4)	−0.2094 (3)	0.07364 (13)	0.0508 (7)	
O2	0.2377 (6)	−0.0151 (4)	0.00790 (14)	0.0709 (10)	
H2	0.2653	−0.1043	−0.0155	0.106*	
O3	0.7077 (5)	0.2844 (3)	0.08218 (13)	0.0568 (8)	
O4	0.7667 (5)	0.4795 (4)	0.01516 (15)	0.0689 (10)	
H4O	0.7958	0.3911	−0.0083	0.103*	
N1	0.1813 (5)	0.2239 (4)	0.23401 (15)	0.0422 (8)	
N2	0.6430 (4)	0.7119 (4)	0.23511 (15)	0.0414 (8)	
C1	0.1727 (5)	0.0818 (4)	0.18659 (17)	0.0341 (8)	
C2	0.1832 (5)	0.0971 (5)	0.11768 (18)	0.0382 (8)	
C3	0.1939 (6)	0.2639 (5)	0.1002 (2)	0.0496 (10)	
H3	0.2005	0.2783	0.0551	0.059*	
C4	0.1951 (6)	0.4099 (5)	0.1488 (2)	0.0521 (11)	
H4	0.1978	0.5234	0.1372	0.062*	
C5	0.1921 (6)	0.3809 (5)	0.2150 (2)	0.0485 (10)	
H5	0.1981	0.4787	0.2486	0.058*	
C6	0.1937 (6)	−0.0559 (5)	0.06520 (18)	0.0413 (9)	
C7	0.1766 (5)	−0.0719 (5)	0.30494 (18)	0.0380 (8)	
C8	0.3176 (6)	−0.1484 (5)	0.34292 (19)	0.0490 (10)	
H8	0.4002	−0.2190	0.3214	0.059*	
C9	0.3357 (7)	−0.1189 (7)	0.4143 (2)	0.0656 (13)	
H9	0.4306	−0.1709	0.4403	0.079*	
C10	0.2159 (8)	−0.0149 (7)	0.4461 (2)	0.0738 (15)	
H10	0.2290	0.0048	0.4936	0.089*	
C11	0.0762 (7)	0.0605 (6)	0.4077 (2)	0.0665 (14)	
H11	−0.0053	0.1320	0.4295	0.080*	
C12	0.0542 (6)	0.0326 (5)	0.3379 (2)	0.0509 (10)	
H12	−0.0426	0.0836	0.3125	0.061*	
C13	0.6743 (5)	0.5728 (5)	0.19104 (18)	0.0357 (8)	
C14	0.6895 (5)	0.5891 (5)	0.12249 (18)	0.0373 (8)	
C15	0.6724 (6)	0.7539 (5)	0.1026 (2)	0.0457 (10)	
H15	0.6818	0.7686	0.0577	0.055*	
C16	0.6416 (6)	0.8959 (5)	0.1483 (2)	0.0463 (10)	
H16	0.6317	1.0081	0.1358	0.056*	
C17	0.6261 (5)	0.8659 (5)	0.2134 (2)	0.0442 (9)	
H17	0.6017	0.9612	0.2444	0.053*	
C18	0.7230 (6)	0.4371 (5)	0.07237 (18)	0.0416 (9)	
C19	0.7107 (5)	0.4283 (5)	0.31237 (18)	0.0380 (8)	
C20	0.5879 (6)	0.3405 (6)	0.3442 (2)	0.0492 (10)	
H20	0.4968	0.2604	0.3184	0.059*	
C21	0.5999 (7)	0.3716 (7)	0.4149 (2)	0.0658 (13)	
H21	0.5161	0.3128	0.4363	0.079*	
C22	0.7329 (8)	0.4871 (7)	0.4529 (2)	0.0717 (14)	
H22	0.7415	0.5066	0.5004	0.086*	
C23	0.8545 (7)	0.5748 (6)	0.4213 (2)	0.0683 (14)	
H23	0.9445	0.6552	0.4476	0.082*	
C24	0.8463 (6)	0.5467 (6)	0.3516 (2)	0.0528 (11)	
H24	0.9308	0.6064	0.3308	0.063*	

### Atomic displacement parameters (Å^2^)

**Table:**

	*U*^11^	*U*^22^	*U*^33^	*U*^12^	*U*^13^	*U*^23^
S1	0.0630 (7)	0.0289 (5)	0.0303 (5)	0.0032 (4)	0.0088 (4)	0.0027 (4)
S2	0.0728 (7)	0.0313 (5)	0.0326 (5)	0.0075 (5)	0.0147 (5)	0.0060 (4)
O1	0.092 (2)	0.0293 (15)	0.0329 (15)	0.0027 (13)	0.0166 (14)	0.0041 (12)
O2	0.148 (3)	0.0337 (16)	0.0387 (17)	0.0088 (18)	0.0413 (19)	0.0042 (13)
O3	0.111 (2)	0.0291 (15)	0.0366 (16)	0.0060 (14)	0.0284 (15)	0.0063 (12)
O4	0.137 (3)	0.0358 (16)	0.0428 (18)	0.0118 (18)	0.0420 (19)	0.0057 (13)
N1	0.062 (2)	0.0298 (17)	0.0348 (18)	0.0000 (14)	0.0090 (15)	0.0022 (14)
N2	0.056 (2)	0.0325 (17)	0.0378 (18)	0.0111 (14)	0.0137 (15)	0.0047 (14)
C1	0.041 (2)	0.0320 (19)	0.0291 (19)	0.0071 (15)	0.0034 (15)	0.0037 (15)
C2	0.054 (2)	0.0300 (19)	0.0309 (19)	0.0066 (16)	0.0075 (16)	0.0034 (15)
C3	0.078 (3)	0.034 (2)	0.040 (2)	0.0069 (19)	0.016 (2)	0.0065 (18)
C4	0.084 (3)	0.028 (2)	0.048 (3)	0.0085 (19)	0.016 (2)	0.0099 (18)
C5	0.068 (3)	0.029 (2)	0.046 (2)	0.0008 (18)	0.013 (2)	−0.0059 (18)
C6	0.065 (3)	0.032 (2)	0.029 (2)	0.0089 (17)	0.0106 (17)	0.0056 (16)
C7	0.053 (2)	0.0293 (19)	0.031 (2)	−0.0026 (16)	0.0100 (17)	0.0019 (15)
C8	0.063 (3)	0.048 (2)	0.038 (2)	0.005 (2)	0.0113 (19)	0.0104 (19)
C9	0.077 (3)	0.080 (3)	0.041 (3)	−0.003 (3)	0.003 (2)	0.021 (2)
C10	0.101 (4)	0.087 (4)	0.032 (2)	−0.020 (3)	0.020 (3)	0.000 (3)
C11	0.087 (4)	0.060 (3)	0.054 (3)	−0.001 (3)	0.035 (3)	−0.010 (2)
C12	0.063 (3)	0.048 (2)	0.045 (2)	0.004 (2)	0.020 (2)	0.0043 (19)
C13	0.044 (2)	0.0323 (19)	0.0295 (19)	0.0029 (15)	0.0065 (15)	0.0008 (15)
C14	0.052 (2)	0.0310 (19)	0.0305 (19)	0.0016 (16)	0.0122 (16)	0.0042 (15)
C15	0.067 (3)	0.032 (2)	0.041 (2)	0.0050 (18)	0.0151 (19)	0.0080 (17)
C16	0.064 (3)	0.032 (2)	0.048 (2)	0.0099 (18)	0.016 (2)	0.0126 (18)
C17	0.060 (2)	0.030 (2)	0.043 (2)	0.0088 (17)	0.0143 (18)	−0.0001 (17)
C18	0.063 (3)	0.032 (2)	0.032 (2)	0.0039 (17)	0.0154 (18)	0.0047 (17)
C19	0.052 (2)	0.033 (2)	0.0301 (19)	0.0079 (16)	0.0102 (16)	0.0056 (16)
C20	0.056 (3)	0.053 (3)	0.039 (2)	−0.001 (2)	0.0074 (19)	0.0115 (19)
C21	0.074 (3)	0.087 (4)	0.044 (3)	0.006 (3)	0.023 (2)	0.024 (3)
C22	0.093 (4)	0.090 (4)	0.033 (2)	0.012 (3)	0.014 (3)	0.005 (3)
C23	0.079 (3)	0.072 (3)	0.046 (3)	0.004 (3)	−0.008 (2)	0.001 (2)
C24	0.064 (3)	0.053 (3)	0.042 (2)	−0.004 (2)	0.009 (2)	0.009 (2)

### Geometric parameters (Å, °)

**Table:**

S1—C1	1.771 (4)	C9—C10	1.360 (7)
S1—C7	1.773 (4)	C9—H9	0.9300
S2—C13	1.769 (4)	C10—C11	1.365 (7)
S2—C19	1.776 (4)	C10—H10	0.9300
O1—C6	1.228 (4)	C11—C12	1.365 (6)
O2—C6	1.306 (4)	C11—H11	0.9300
O2—H2	0.8200	C12—H12	0.9300
O3—C18	1.218 (4)	C13—C14	1.411 (5)
O4—C18	1.308 (4)	C14—C15	1.379 (5)
O4—H4O	0.8200	C14—C18	1.478 (5)
N1—C5	1.315 (5)	C15—C16	1.367 (5)
N1—C1	1.334 (4)	C15—H15	0.9300
N2—C17	1.315 (5)	C16—C17	1.369 (5)
N2—C13	1.333 (4)	C16—H16	0.9300
C1—C2	1.409 (5)	C17—H17	0.9300
C2—C3	1.374 (5)	C19—C20	1.376 (5)
C2—C6	1.472 (5)	C19—C24	1.386 (5)
C3—C4	1.374 (5)	C20—C21	1.386 (5)
C3—H3	0.9300	C20—H20	0.9300
C4—C5	1.372 (5)	C21—C22	1.354 (7)
C4—H4	0.9300	C21—H21	0.9300
C5—H5	0.9300	C22—C23	1.366 (7)
C7—C8	1.372 (5)	C22—H22	0.9300
C7—C12	1.387 (5)	C23—C24	1.371 (6)
C8—C9	1.397 (5)	C23—H23	0.9300
C8—H8	0.9300	C24—H24	0.9300
			
C1—S1—C7	103.18 (16)	C11—C12—C7	119.9 (4)
C13—S2—C19	103.20 (16)	C11—C12—H12	120.1
C6—O2—H2	109.5	C7—C12—H12	120.1
C18—O4—H4O	109.5	N2—C13—C14	121.1 (3)
C5—N1—C1	118.8 (3)	N2—C13—S2	117.3 (3)
C17—N2—C13	118.6 (3)	C14—C13—S2	121.6 (3)
N1—C1—C2	121.4 (3)	C15—C14—C13	117.9 (3)
N1—C1—S1	116.8 (3)	C15—C14—C18	119.8 (3)
C2—C1—S1	121.8 (3)	C13—C14—C18	122.3 (3)
C3—C2—C1	117.6 (3)	C16—C15—C14	120.5 (4)
C3—C2—C6	119.1 (3)	C16—C15—H15	119.8
C1—C2—C6	123.1 (3)	C14—C15—H15	119.8
C2—C3—C4	120.7 (4)	C15—C16—C17	117.2 (3)
C2—C3—H3	119.6	C15—C16—H16	121.4
C4—C3—H3	119.6	C17—C16—H16	121.4
C5—C4—C3	117.1 (4)	N2—C17—C16	124.7 (3)
C5—C4—H4	121.5	N2—C17—H17	117.6
C3—C4—H4	121.5	C16—C17—H17	117.6
N1—C5—C4	124.3 (3)	O3—C18—O4	122.1 (3)
N1—C5—H5	117.8	O3—C18—C14	123.5 (3)
C4—C5—H5	117.8	O4—C18—C14	114.3 (3)
O1—C6—O2	121.9 (3)	C20—C19—C24	119.4 (4)
O1—C6—C2	123.8 (3)	C20—C19—S2	117.8 (3)
O2—C6—C2	114.2 (3)	C24—C19—S2	122.5 (3)
C8—C7—C12	119.6 (4)	C19—C20—C21	120.0 (4)
C8—C7—S1	117.3 (3)	C19—C20—H20	120.0
C12—C7—S1	122.9 (3)	C21—C20—H20	120.0
C7—C8—C9	119.3 (4)	C22—C21—C20	120.4 (4)
C7—C8—H8	120.3	C22—C21—H21	119.8
C9—C8—H8	120.3	C20—C21—H21	119.8
C10—C9—C8	120.6 (5)	C21—C22—C23	119.7 (4)
C10—C9—H9	119.7	C21—C22—H22	120.2
C8—C9—H9	119.7	C23—C22—H22	120.2
C9—C10—C11	119.6 (4)	C22—C23—C24	121.3 (4)
C9—C10—H10	120.2	C22—C23—H23	119.3
C11—C10—H10	120.2	C24—C23—H23	119.3
C10—C11—C12	121.1 (4)	C23—C24—C19	119.3 (4)
C10—C11—H11	119.5	C23—C24—H24	120.4
C12—C11—H11	119.5	C19—C24—H24	120.4
			
C5—N1—C1—C2	2.8 (5)	C17—N2—C13—C14	0.1 (5)
C5—N1—C1—S1	−177.9 (3)	C17—N2—C13—S2	178.6 (3)
C7—S1—C1—N1	−7.2 (3)	C19—S2—C13—N2	−8.3 (3)
C7—S1—C1—C2	172.1 (3)	C19—S2—C13—C14	170.2 (3)
N1—C1—C2—C3	−2.9 (6)	N2—C13—C14—C15	0.4 (6)
S1—C1—C2—C3	177.8 (3)	S2—C13—C14—C15	−177.9 (3)
N1—C1—C2—C6	173.5 (3)	N2—C13—C14—C18	−179.6 (3)
S1—C1—C2—C6	−5.8 (5)	S2—C13—C14—C18	2.0 (5)
C1—C2—C3—C4	0.2 (6)	C13—C14—C15—C16	0.0 (6)
C6—C2—C3—C4	−176.3 (4)	C18—C14—C15—C16	−179.9 (4)
C2—C3—C4—C5	2.4 (6)	C14—C15—C16—C17	−1.0 (6)
C1—N1—C5—C4	0.1 (6)	C13—N2—C17—C16	−1.3 (6)
C3—C4—C5—N1	−2.6 (7)	C15—C16—C17—N2	1.7 (6)
C3—C2—C6—O1	−171.7 (4)	C15—C14—C18—O3	−166.9 (4)
C1—C2—C6—O1	12.0 (6)	C13—C14—C18—O3	13.2 (6)
C3—C2—C6—O2	8.6 (6)	C15—C14—C18—O4	11.6 (6)
C1—C2—C6—O2	−167.8 (4)	C13—C14—C18—O4	−168.3 (4)
C1—S1—C7—C8	−122.4 (3)	C13—S2—C19—C20	126.3 (3)
C1—S1—C7—C12	62.3 (4)	C13—S2—C19—C24	−59.6 (4)
C12—C7—C8—C9	−0.2 (6)	C24—C19—C20—C21	0.3 (6)
S1—C7—C8—C9	−175.7 (3)	S2—C19—C20—C21	174.6 (3)
C7—C8—C9—C10	−0.3 (7)	C19—C20—C21—C22	−0.5 (7)
C8—C9—C10—C11	0.3 (8)	C20—C21—C22—C23	0.7 (8)
C9—C10—C11—C12	0.3 (8)	C21—C22—C23—C24	−0.8 (8)
C10—C11—C12—C7	−0.8 (7)	C22—C23—C24—C19	0.7 (7)
C8—C7—C12—C11	0.8 (6)	C20—C19—C24—C23	−0.4 (6)
S1—C7—C12—C11	176.0 (3)	S2—C19—C24—C23	−174.4 (3)

### Hydrogen-bond geometry (Å, °)

**Table:**

*D*—H···*A*	*D*—H	H···*A*	*D*···*A*	*D*—H···*A*
O2—H2···O3^i^	0.82	1.82	2.624 (2)	168 (1)
O4—H4o···O1^i^	0.820	1.83000	2.642 (2)	169.62 (19)
C3—H3···O4^ii^	0.93	2.50	3.264 (5)	139
C4—H4···O1^iii^	0.93	2.55	3.458 (5)	164
C15—H15···O2^ii^	0.93	2.54	3.294 (5)	138
C16—H16···O3^iii^	0.93	2.58	3.467 (5)	160

Symmetry codes: (i) −*x*+1, −*y*, −*z*; (ii) −*x*+1, −*y*+1, −*z*; (iii) *x*, *y*+1, *z*.
